# Nanoparticles in Sentinel Lymph Node Assessment in Breast Cancer

**DOI:** 10.3390/cancers2041884

**Published:** 2010-11-17

**Authors:** Laura Johnson, Geoff Charles-Edwards, Michael Douek

**Affiliations:** 1 Department of Research Oncology, Kings College London, 3rd Floor Bermondsey Wing, Guy’s Hospital, London, SE1 9RT, UK; E-Mail: laura.johnson@kcl.ac.uk; 2 Department of Medical Physics, Guys and St Thomas’ NHS Foundation Trust, 4th Floor Lambeth Wing, St Thomas’ Hospital, London, SE1 7EH, UK; E-Mail: geoff.charles-edwards@kcl.ac.uk

**Keywords:** axillary staging, breast cancer, nanoparticles, sentinel lymph node

## Abstract

The modern management of the axilla in breast cancer relies on surgery for accurate staging of disease and identifying those patients at risk who would benefit from adjuvant chemotherapy. The introduction of sentinel lymph node biopsy has revolutionized axillary surgery, but still involves a surgical procedure with associated morbidity in many patients with no axillary involvement. Nanotechnology encompasses a broad spectrum of scientific specialities, of which nanomedicine is one. The potential use of dual-purpose nanoprobes could enable imaging the axilla simultaneous identification and treatment of metastatic disease. Whilst most applications of nanomedicine are still largely in the laboratory phase, some potential applications are currently undergoing clinical evaluation for translation from the bench to the bedside. This is an exciting new area of research where scientific research may become a reality.

## 1. Introduction

The assessment and management of the axilla in breast cancer is a key factor in defining prognosis and determining the need for adjuvant chemotherapy. To date, there is no readily available, cheap, non-invasive, reliable, and most of all safe method of imaging metastatic spread from a primary breast neoplasm to the ipsilateral axilla.

Modern magnetic resonance imaging (MRI) is rapidly advancing and now synchronous, dynamic breast imaging can be undertaken rapidly with a high spatial resolution. However, advances in MRI and computed tomography (CT) are limited by the drawbacks of non-specific contrast agents. These agents are usually administered systemically causing changes that are more wide spread when localized identification is what is actually required. They cause rare but significant systemic toxicity with the risk of end organ damage particularly with the common iodine based substrates, a small risk a clinician has to assess on a patient-by-patient basis. Nanomedicine heralds a wave of new agents termed theranostic particles that can address these issues and may offer the hope of synchronous diagnosis and treatment, in the future. Most research in this area is still at the bench side and clinical translation is restricted by toxicity and particle instability.

## 2. History of the Sentinel Lymph Node

Sentinel lymph node biopsy (SLNB) is now the gold standard in staging the axilla in breast cancer. The sentinel lymph node (SLN) was first described by Cabanas whilst undertaking penile lymphangiography for cancer, one node was consistently identified to receive lymphatic flow. This was histologically confirmed to be the first, and in some cases the only site, of metastatic spread of penile cancer [1]. The technique of SLNB, however, was not popularized until 1992 when Morton reported SLN identification for the staging of cutaneous malignant melanoma using patent blue dye [2]. This was quickly translated to the staging of the axilla in breast cancer by Giuliano *et al*. injecting isosulphan blue dye peritumorally in the affected breast [3]. SLNB relies on the observation that the sentinel node(s) are the first and most likely place for lymph node metastasis and reliably reflect the likely presence of further metastases in the axillary basin. In breast cancer, identification and histological examination of the SLN should identify those patients with an involved SLN who require further surgery in the form of an axillary lymph node dissection (ALND), whilst sparing those with a normal SLN the morbidity of axillary node clearance. Giuliano’s first reported success rate in identifying the SLN was 66% and correct prediction of the status of the axillary node basin was 96%. In part, this initial study optimized the technique for SLNB and included many cases that are now considered inappropriate for SLNB, for example patients with overt metastatic nodal involvement. By 1994, in the same experienced Institute, a subsequent publication demonstrated SLN identification in 97% using blue dye alone [4]. Interest was growing in the SLNB and in parallel to other work on cutaneous melanoma [5], Veronesi and others highlighted the drawback of blind dissection for a blue lymph node and demonstrated SLN detection using a radioactive tracer (Technetium-99m labeled sulfur colloid) and a hand held gamma probe [6]. Pre-operative lymphoscintigraphy, in addition to intra-operative identification using a gamma probe, successfully identified the SLN in 85–96% of patients [6,7]. Subsequently, larger studies have shown that a combination of a blue dye with radioactive tracer improved detection rates for SLNB to greater than 90% with a false negative rate of less than 5% [8]. In view of this very high SLN detection rate, and the poor spatial resolution of lymphoscintigraphy, many centers no longer perform pre-operative lymphoscintigraphy.

SLNB causes significantly less morbidity than ALND (lower risk of nerve injury, lymphoedema, injury to axillary vein and shoulder stiffness) and requires a shorter hospital stay [9,10]. It is nevertheless an invasive procedure with complications and is usually performed under general anesthesia. It assesses only the SLN (not the entire axillary basin), has a false-negative rate of at least 5% in experienced hands and does not take into account the presence of skip lesions [11]. The Achilles’ heel of SLNB is a macroscopically involved lymph node that would block the transition of the tracer or blue dye and result in technical failure of identification of the involved node, because of the presence of collateral lymphatic drainage. Furthermore, in excess of 50% of patients who undergo a SLNB will have a negative node following excision [12]. With improved imaging techniques, these patients could, in future, be spared an unnecessary operation.

## 3. Alternatives to SLNB for Staging of the Axilla in Breast Cancer

Lymph node assessment in breast cancer can be considered in one of two ways. Firstly, pre-operative (non-invasive) evaluation of the lymphatic basin to identify or exclude metastases, and secondly, intraoperative identification of the nodes enabling histological examination to exclude or confirm metastatic disease. Various imaging modalities have been used to image the SLN successfully.

Ultrasound (US) of the axilla is now used routinely in patients with breast cancer. It is cheap, non invasive, acceptable to patients and readily available. Sonographic criteria for selecting indeterminate, suspicious, or metastatic-appearing lymph nodes are a thickened cortex, lobulation of the cortex, reduction or loss of hilar fat when compared with other ipsilateral or contralateral lymph nodes [13]. Pre-operative ultrasound-guided fine needle aspiration cytology (FNAC) can identify patients who require axillary lymph node dissection (ALND) and who are thus not suitable for SLNB. Identification rates for axillary metastases vary and reflect operator variability between units, but in a recent report 37% of patients with an involved axilla were identified pre-operatively with US and FNAC [14]. Axillary ultrasound was more sensitive in patients with symptomatic cancers than those who were screen detected. A smaller series mirrors these findings demonstrating 58% sensitivity and 100% specificity for pre-operative identification of an involved axilla using US and FNAC [15].

Positron emission tomography (PET) integrated with computed tomography (CT) scanning (PET/CT) could successfully identify preoperatively axillary involvement in patients with breast cancer. Almost 20% of patients following PET scanning can be spared SLNB and undergo ALND as the primary axillary surgery with 77.1% sensitivity and 100% specificity [16]. PET/CT is, however costly, exposes patients to radiation and is often not readily available in many cancer centers.

Immunoscintigraphy using a technetium^99m^ labeled murine monoclonal antibody is known to identify a range of human adenocarcinomas (Thomsen-Friedenreich (TF) antigen) and can demonstrate the presence of axillary metastases in breast cancer following intravenous injection (sensitivity 71%, specificity 89%) [17]. This involves the use of a radioactive tracer associated with strict legislation and exposes the patient and the staff to small doses of ionizing radiation. It is in part due to these drawbacks that alternative techniques for lymph node imaging are being sought.

## 4. Nanoparticles

Nanoparticles offer the hope of overcoming many of the challenges involved with breast cancer staging. These include the use of toxic, non-specific, systemically administered contrast agents, limited availability of specialized imaging modalities such as CT/PET, and the operator variability and inter-observer error in identifying axillary metastases.

Nanotechnology is a field of science first brought into the public domain by Feynman in his lectures in 1959 [18], and he was later awarded the Nobel Prize in Physics in 1965. It holds great promise in medicine, physics, chemistry and engineering alike. A nano particle ranges in size from 1 to 100 nanometers. One nanometer is one billionth of a meter, or in real terms; 100,000 times smaller than a human hair. The potential clinical applications of nanoparticles are immense, in particular in their innovative approach to cancer diagnosis and therapy [19].

The behavior of nanoparticles depends upon their size and charge. Smaller particles, whilst undergoing renal filtration and clearance, will also undergo extravasation into the surrounding tissue when flowing through 'leaky' vessels. This property is ideal for the accumulation of particles that extravasate through leaky neoangiogenic blood vessels, typically found in cancer. This effect is known as enhanced permeability and retention effect (EPR) [20]. Larger lymphotropic nanoparticles are identified by the host immunological system and undergo phagocytosis with uptake into lymphoid cells. Here the nanoparticles with the appropriate surface coating are 'trapped' within the lymph node where they can be identified by numerous methods and differentiate normal from abnormal lymph node architecture [7]. A nanoparticle platform combined with various different modalities attached to its surface including chemotherapeutic drugs and imaging contrast agents have the capability to integrate cancer biology, diagnostic imaging and treatment in one. The addition of a specific modality attached to a theranostic particle with an appropriate size and charge would allow directed migration of the particle through the body with concentration at the site of action with maximum result and minimal systemic effect [21]. A great amount of work is being undertaken in this field, although at present much remains at the bench side, with translation to the clinic limited by FDA approval, toxicity and availability of resources [19,22]. There are a number of current clinical trials to evaluate assessment of the axilla in breast cancer and other exciting laboratory work with specific challenges that prevent translation from the bench to the bedside.

### 4.1. Pre-Operative (Non-Invasive) Staging of the Axillary Sentinel Lymph Node Using Nanoparticles

Super-paramagnetic nanoparticles (SPIOs) have been the focus of much attention for imaging lymph nodes. SPIOs have intrinsic paramagnetic properties only when influenced by an external magnetic field, avoiding undesirable magnetic agglomeration. Following intravenous administration, SPIOs are transported to lymphatic tissue where they have a negative (darkening) effect on MRI with T2 and T2*-weighted imaging protocols. Non-homogenous uptake of contrast in the SLN may help identify a metastatic node [23,24]

Ultra small superparamagnetic iron oxide nanoparticles (USPIOs, <50 nm) have been used in humans as an MRI contrast agent, injected intravenously (IV), to assess the axilla. Using a 1.5 T MRI scanner, involved axillary lymph nodes can be identified 24 hours post-injection, in breast cancer patients with a sensitivity of 82% and 100% specificity [25]. Koh characterized three patterns of lymph nodes on MRI following administration of USPIOs [26]. These are demonstrated in Table 1. One group performed axillary staging with MRI after IV USPIO injection in addition to gadolinium and demonstrated superior results to USPIO injection alone, especially on a T1 gradient echo with fat saturation [27]. When USPIO/ gadolinium enhanced MRI was combined with 18F-fluorodeoxyglucose positron emission tomography (FDG-PET) there was 100% sensitivity and specificity (n = 10) for identifying a metastatic axillary lymph node. These results are very promising but the number of cases is far too small to change current practice.

**Table 1:** Grading of node appearance on MRI following USPIO administration [26].

Group	Description
**1**	Normal morphology with uniform or central signal drop (categorized as normal)
**2**	Normal morphology without or with partial signal drop (categorized as partial or total invasion)
**3**	Focal or global volume increase without or with partial signal drop (characterized as partial or total invasion

### 4.2. Intraoperative Identification of the Axillary SLN

**Figure 1:**
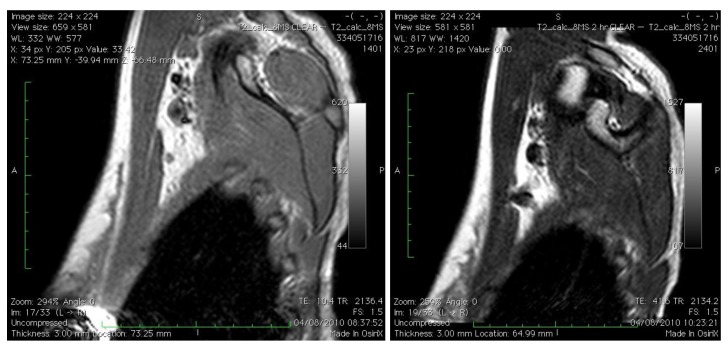
Left panel: right axilla pre-Endorem injection on MRI scans; right panel: SLN easily identified 120 minutes post-Endorem injection intradermally.

Using an SPIO (Endorem, Guerbet, Paris) injected directly into the breast of women with breast cancer before surgery we have demonstrated the position and morphological appearance of the SLN using MRI scanning (Figure 1) in addition to subsequent intraoperative SLN identification using a hand-held magnetometer (SentiMag, Endomagnetics, UK). Successful identification of the SLN using this technique compared to blue dye and technetium^99^ was successful in 100% of patients in an initial pilot study [28]. Using SPIO, the SLN is identified with visual inspection of the node (black staining) in addition to localization with a hand-held magnetometer. This technique is a viable, reproducible, non-invasive and non-radioactive method of SLN assessment with successful intraoperative identification.

Gold (Au) nanocages (150 nm) have successfully been demonstrated in a rat model, for localization of the SLN following intradermal injection. Identification of the SLN was successful using a photo-acoustic ultrasonic transducer and a 10 Hz pulse-repetition-rate laser system over increasing tissue depths up 33 mm below the skin surface, depths akin to the average depth of the axilla SLN in humans (12 mm ± 5 mm) [29]. There was also dark staining of the SLN, from substrate accumulation in the subcapsular sinus aiding visual identification. At 140 minutes post-contrast injection, peak accumulation of Au-nanocages were seen in the lymph node at concentrations in excess of triple that injected. No comment was made as to the distribution of the nanocages throughout the body and/or its toxicity. The study is limited to animal models until FDA approval for Au nanocages is granted. Although gold is currently licensed for use in human therapy [30], however, toxicity of injection site and lymph node accumulations of nanocages, may limit clinical application.

Surguladze *et al*. presented ‘UNIMAG’, an iron-oxide nanoparticle, which when injected peritumorally was transported to the SLN and taken up by macrophages resulting in black staining of lymph nodes [31]. They hypothesized future applications of imaging pre-and intraoperatively to more accurately identify the SLN. No comment was made as to the primary tumor in question or the number of cases studied, however, this principle would easily translate to SLN identification in breast cancer.

Micro bubbles—not strictly a nanoparticle being >100 nm in size (200–500 nm)—but still measurable using nanometers, have been successfully demonstrated to identify the SLN in women with breast cancer. The method of microbubble injection subcutaneously into the breast enhances the SLN on US imaging. This does not aim to differentiate involved from uninvolved SLNs based on morphology but aims to localize the SLN. The drawback is a very short contrast enhancement lasting several minutes. Sever *et al*. placed a guide wire in the SLN pre-operatively and the node excised intraoperatively. The microbubble enhancement technique was successful in identifying the sentinel node(s) in 89% of patients, and five patients who had an involved sentinel node, ultrasound successfully detected all cases (100%) [32]. Another clinical application could be pre-operative needle core-biopsy (NCB) of the SLN in clinic under local anesthetic. Clearly this would be limited by the drawback of NCB, since a biopsy may not be representative of the entire node.

In a mouse model, fluorescent silica nanoparticles demonstrated axillary lymph nodes with some success. Fluorescence particles themselves pass too readily through the SLN, but embedding them in a silica nanoparticle matrix results in entrapment within the node. Detection *in vivo* using a fluorescence imaging system five minutes post intradermal injection was only possible following skin excision overlying the axilla. The biodistribution of these nanoparticles showed preferential uptake into the SLN over other organs and previous work has showed the nanoparticle in mice appears to be non toxic, however, a lack of transcutaneous visualization at this time limits potential clinical use [33,34]

Quantum dots (QDs) are fluorescent inorganic nanometer sized crystals. A semi-conductor core of a heavy metal is encapsulated by an organic ‘shell’ and this is coated with polymeric or lipid based layers. The later is to minimize toxic effects following oxidization of the core metals. In mouse models, intradermal injection with QDs identifies the axillary SLN in under three minutes [35] and remains within the SLN for a period of over 24 hours before migrating further to higher echelon nodes in the lymphatic basis. Toxicity and biodistribution studies confirm QDs are not renally cleared and concentrate predominantly at the injection site and within the SLN. It was initially thought that following dorsal flank injection, QDs, in time, may accumulate in the liver and spleen among other organs [36]. However, when injected into the mouse paw, more closely mimicking the anatomy for breast sentinel node biopsy, they do not appear to distribute to other organs within the body [37]. Akin to silica nanoparticles, fluorescence, however, is only appreciated in the SLN following removal of axillary skin. Using near infra red (NIR) emitting QDs that more deeply penetrate tissue may overcome this hurdle, but the greatest limiting factor to the otherwise ideal nanoparticle translating into the clinical setting, is eliminating the toxicity from the heavy metal whilst maintaining particle stability.

Immunoglobulin (Ig)-conjugated NIR optical probes behave in a very similar way to QDs in their pharmacokinetics and ability to identify the axillary SLN. In a mouse model following intra-dermal injection into the breast pad, fluorescence was seen on the node surface in just one minute and is retained in the axilla for 30 minutes allowing sufficient time for SLN excision. Since these probes are derived from immunoglobulins, they are recognized by the host immune system and concentrate preferentially in the medulla of the node and are more likely to be retained for a longer period, whereas QDs enhance both the cortex and the medulla of the node equally [38]. Toxicity studies are yet to be undertaken before clinical studies can begin.

Dendrimers are small nanoparticles (less than 15 nm in size) composed of highly branched synthetic polymers, which are gaining increasing popularity. Dendrimers have ‘space’ within the core which can harbor smaller particles (e.g., contrast agent particles), are non-immunogenic, and have a prolonged circulation half-life [39]. Dendrimers have been utilized to bind MRI contrast agents (Gadolinium) in addition to an NIR fluorophore, enabling pre-operative 3D identification of the axillary lymph nodes in mice using MRI and intra-operative visualization of the SLN with an optical imaging unit. Intraoperative visualization of the fluorophore can penetrate skin up to a depth of 2 cm, making it suitable for clinical use. This single injection-dual purpose nanoparticle remains identifiable for both imaging modalities in the SLN for at least two hours post injection [40]. This does not characterize the lymph node, but more reliably identifies its location pre and intraoperatively for subsequent excision and histological assessment. Prior to clinical translation, toxicity testing is necessary, but this is not thought to pose a big problem due to minimal systemic absorption.

### 4.3. In vivo Treatment of the Axillary SLN

One novel approach to the SLN in breast cancer is to treat the SLN rather than excise it. In a mouse model, a metastatic SLN has been replicated by injecting breast cancer cells labeled with gold carbon nanotubules (GNT) and fluorescence into in a SLN to replicate a metastatic SLN. This was targeted with a low laser pulse energy, and the GNT-containing breast cancer cells within the SLN deteriorated as demonstrated by loss of fluorescence and transmission images [41]. This would perhaps have clinical application to augment a process of pre-operative assessment of the axillary lymphatics by imaging whereby the SLN deemed ‘normal’ could be ‘treated’, thus eliminating the presence of any micrometastases or isolated tumor cells that may otherwise remain.

## 5. Challenges in the Clinical Translation of Novel Nanoparticles

A large amount of time, effort and resources goes into any research project, but success at the bench may not be so readily seen in clinical practice as the process of translation is challenging. Developing the research protocol is limited by many factors—all which must be overcome before work can begin. These include availability of funding, personnel, health and safety, ethics approval and most important of all, regulatory approval. Since each aspect requires clearance, often independent to the others, there are many barriers that can delay the process. In addition, changes to one aspect to overcome a problem may often have an impact on another, and the whole process must begin again.

Once the research element is complete and the evidence is available to suggest a change in practice or a new technique, further hurdles remain. Changes to practice are slow and often only partially adopted, if adopted at all into routine clinical care [42], and a specific framework should be in place to ease implementation [43]. There is also the consideration for cost analysis and benefit ratio. On initial impression, it may seem that a single MRI scan with contrast agent may be cheaper than the cost of an operation and subsequent hospital stay. However, with potential increasing availability, popularity and success of imaging modalities their utilization may be more widespread, in turn increasing expenditure. A clear benefit both from a cost and a patient perspective must be identified and advertised as part of the implementation process. For novel device and nanoparticle research, there is a need to ensure clinical involvement at an early stage in order to expedite the translation of promising new clinical tools from the bench to the bedside.

## 6. Conclusions

Nanotechnology has been described as the ‘small technology with a big impact’. As our understanding of cellular processes and individual cancer cell fingerprints increases, the scope for more targeted tissue specific nanoparticles is endless. Most nanoparticles applied to medicine are biocompatible, with the exception of QDs, and are metabolized via the normal biochemical pathways. In most cases, the products of their metabolism are integrated into normal mineral pools within the body for reuse, storage or excretion.

Surgery remains at present the most important modality for axillary node staging in breast cancer to accurately identify those patents that will benefit from adjuvant chemotherapy for the treatment of metastatic disease. In the future, nanotechnology may help to improve patient selection for surgery and in time may indeed identify patients pre-operatively whom do not have axillary disease and spare them surgery to the axilla altogether.

Nanotechnology has started to create multifunctional particles blending diagnosis and treatment together, with the promise of nanomedicine paving the way for better cancer detection, management and treatment. Further research is needed to select the most promising clinical application and expedite translation from the bench to the bedside.
